# Enzymatic reactions in teleocidin B biosynthesis

**DOI:** 10.1007/s11418-021-01504-2

**Published:** 2021-03-06

**Authors:** Takayoshi Awakawa

**Affiliations:** 1 Graduate School of Pharmaceutical Sciences, The University of Tokyo, Bunkyo-ku, Tokyo, 113-0033 Japan; 2 Collaborative Research Institute for Innovative Microbiology, The University of Tokyo, Yayoi 1-1-1, Bunkyo-ku, Tokyo, 113-8657 Japan

**Keywords:** Terpene indole, Biosynthesis, Protein kinase C, X-ray crystal structure analysis

## Abstract

The teleocidin B family members are terpene indole compounds isolated from *Streptomyces* bacteria, and they strongly activate protein kinase C (PKC). Their unique structures have attracted many researchers in the natural product chemistry and pharmacology fields, and numerous isolation and bioactivity studies have been conducted. The accumulated information has facilitated the identification of the enzymatic reactions in teleocidin biosynthesis, and new developments in structural biology have strongly aided efforts to clarify the finer points of these reactions. This review describes the recent biochemical and structural biological studies to reveal their reaction mechanisms, with a primary focus on the terpene cyclization triggered by the C-N bond formation by P450 oxygenase (TleB), the prenyltransferase (TleC), and the methyltransferase (TleD). This new knowledge will benefit future engineering studies to create unnatural PKC activators.

## Introduction

**Fig. 1:**
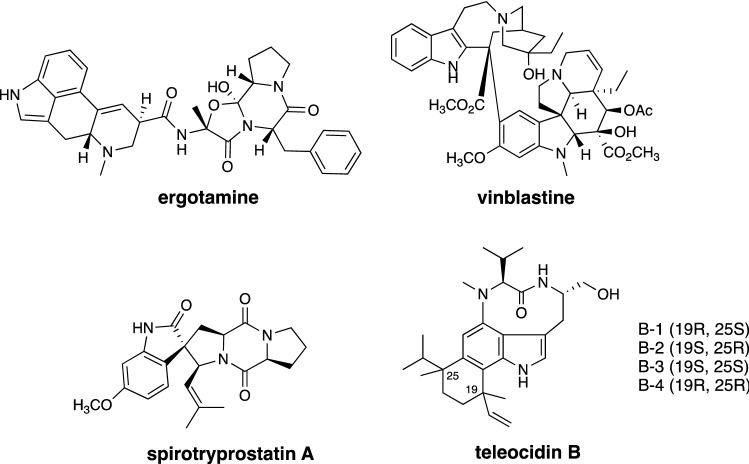
Structures of representative terpene indole alkaloids

**Fig. 2:**
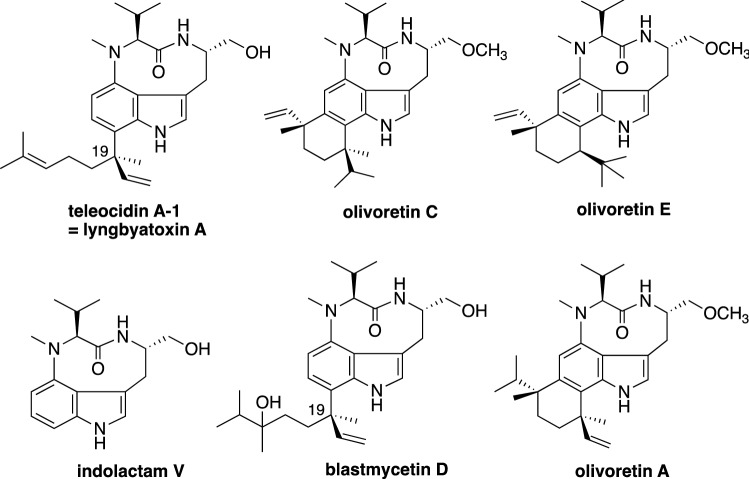
Structures of the isolated teleocidin compounds

Terpene indole compounds have an abundance of structural diversity, derived from the electron-rich indole ring and the cation-generating terpenoid [1–5]. This group includes large numbers of bioactive compounds, including ergotamine (vasoconstrictor), vinblastine (microtubule dynamics inhibitor), spirotryprostatin (antimitotic arrest agent), and teleocidin B (protein kinase C activator) (Fig. 1). The unique structure of teleocidin B and its robust bioactivity in the activation of protein kinase C (PKC) have drawn keen attention from natural product researchers [6]. Numerous teleocidin B analogs have been isolated from natural sources (Fig. 2) since the first compound teleocidin B-4 was identified through NMR and X-ray structural studies [7–10]. The isolated analogs not only furnished a pool of natural medicinal compounds but also provided beneficial information to construct the hypothetical biosynthetic pathways. In addition, isotope-feeding and chemical transformation experiments have generated useful clues for deducing biosynthetic pathways, such as the transformation of blastmycetin D to olivoretin A by acid treatment [11, 12]. The isolation of lyngbyatoxin A (= teleocidin A-1) from the cyanobacterium *Moorea producens* [13], and the establishment of its biosynthetic gene cluster also facilitated the identification of the teleocidin gene cluster [14]. The PKC activators are recognized as tumor growth enhancers, but a recent study has reported that the subtype-specific activation of PKC actually represses tumor growth [15]. Thus, pharmacologically useful analogs are likely to be present in the library of teleocidin analogs. Therefore, we investigated the biosynthesis of teleocidin for the biosynthetic construction of a base for the creation of a library of PKC activators. This review updates the prior reviews on teleocidin biosynthesis [16, 17], and mainly describes the discovery of the gene cluster and the biochemical and structural studies of the biosynthetic enzymes in teleocidin biosynthesis.

## Identification of teleocidin biosynthetic gene cluster (*tle* cluster)

**Fig. 3:**
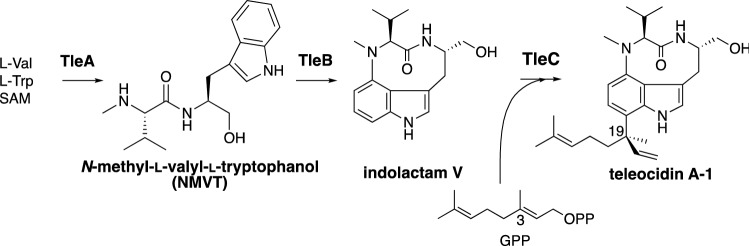
Biosynthetic reactions of lyngbyatoxin A = teleocidin A-1

**Fig. 4:**
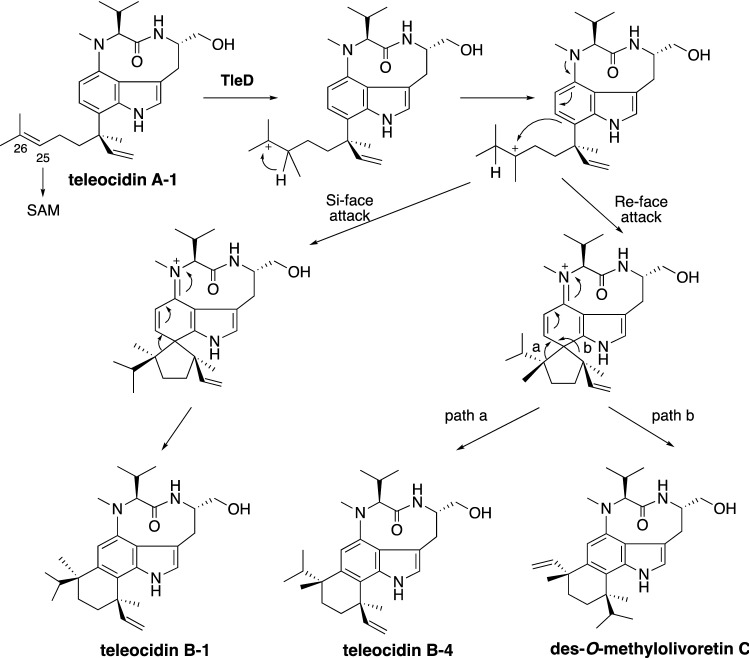
Terpene cyclization triggered by the methylation of teleocidin A-1 by TleD

The biosynthetic gene cluster of teleocidin B (*tle* cluster), which includes genes encoding a non-ribosomal peptide synthetase (NRPS, *tleA*), P450 oxygenase (*tleB*), and prenyltransferase (*tleC*), was found in the genome of a teleocidin B producer, *Streptomyces blastmyceticus*, based on the similarities to lyngbyatoxin biosynthetic enzymes in cyanobacteria [18]. The reactions of TleABC were deduced from the lyngbyatoxin biosynthetic reactions [14, 19, 20], as the synthesis of *N*-methyl-L-valyl-L-tryptophanol (NMVT) by TleA, the C-N bond formation by TleB, and the prenyl transfer by TleC (Fig. 3). We expected that the 23-kb genomic region including the *tle* cluster would be responsible for the production of teleocidin B. Unexpectedly, the heterologous expression of the *tle* cluster in *Streptomyces lividans* yielded only teleocidin A-1 (Fig. 2). Thus, the C-methyltransferase (C-MT) genes encoded outside of the *tle* cluster were screened by co-expressing C-MT genes in addition to the *tle* cluster. As a result, the expression of one of the transcribed C-MT genes (named *tleD*) led to the production of teleocidin B-1, teleocidin B-4, and des-*O*-methylolivoretin C (Fig. 4), indicating that these compounds are derived from a common intermediate and the terpene structure diversifies after C-methylation. The in vitro TleD reaction with [D-25]lyngbyatoxin A and *S*-adenosylmethionine (SAM) produced [D-26]teleocidin B-4, suggesting that the deuterium atom shifts from C-25 to C-26 during the terpene ring cyclization. Finally, we proposed the reaction of TleD: first, C-25 was C-methylated from SAM by TleD, and the resultant cation migrated from C-25 to C-26. Secondly, the cation at C-25 reacted with C-7 of the indole in *Re*-face attack to form the spiro intermediate. The spiro intermediate spontaneously transformed into teleocidin B-4 via path A and des-*O*-methylolivoretin C via path B (Fig. 4). The unfavored *Si*-face attack yielded the spiro intermediate with differently oriented vinyl and isopropyl groups, leading to the minor product, teleocidin B-1. This was the first identification of terpene cyclization triggered by C-methylation in nature.

## Structural analysis of TleB

**Fig. 5:**
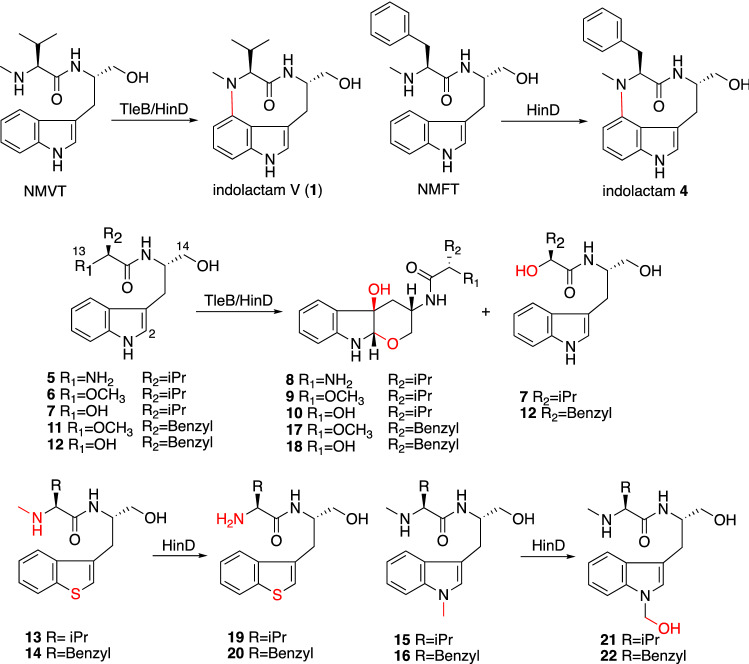
The reactions of TleB and HinD

**Fig. 6:**
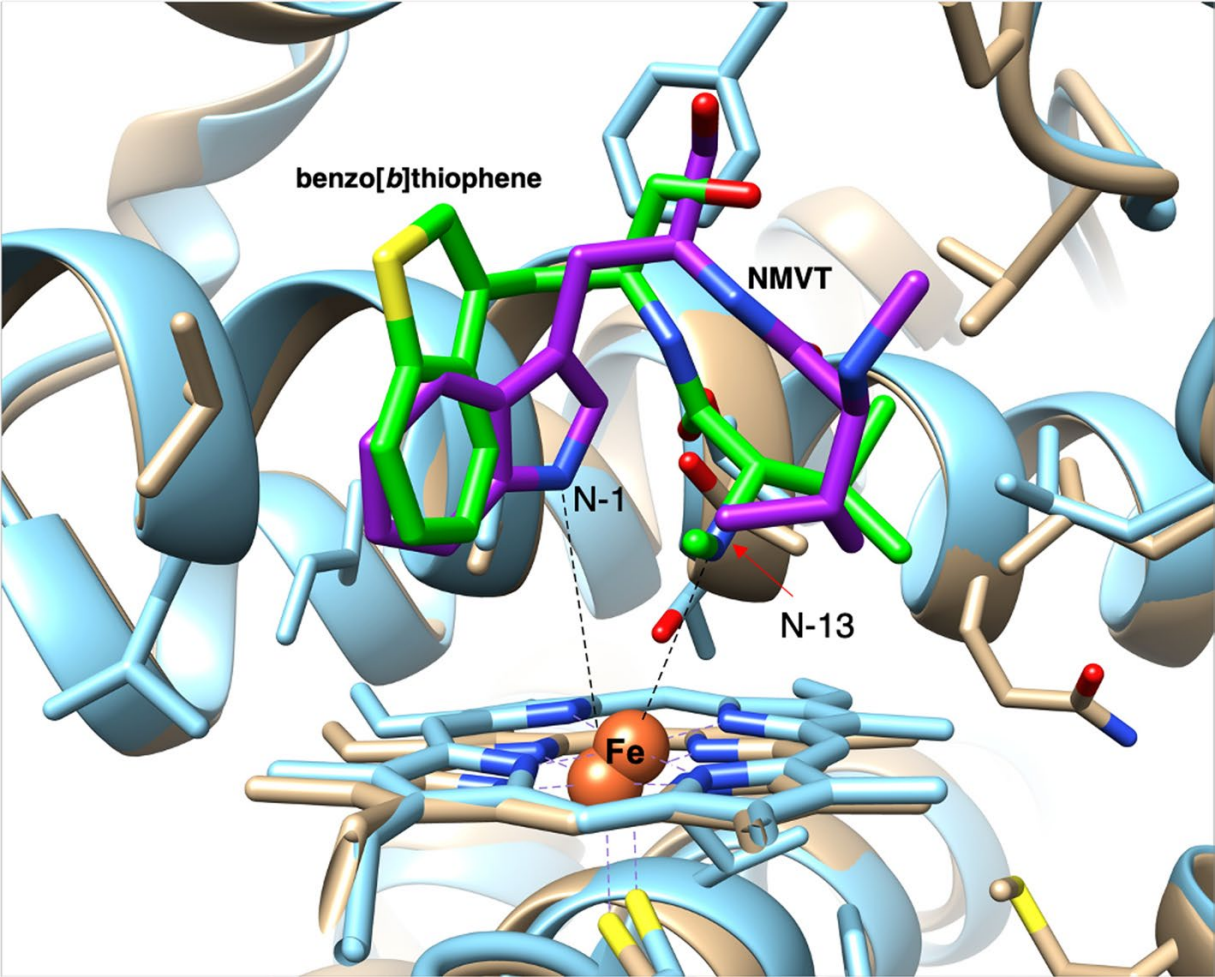
The active site structures of TleB (brown, complexed with NMVT, PDB: 6J83) and HinD (cyan, complexed with the benzo[*b*]thiophene **13**, PDB: 6J88)

The nine-membered indolactam V structure is the most important for PKC activation in teleocidin B [21], and its C-N bond is constructed by a P450 monooxygenase which is a versatile heme–iron protein [22]. A P450 oxygenase encoded in the *tle* cluster, TleB, was deduced to accept NMVT and form the C-N bond between N-13 and C-4 to produce indolactam V, as shown in the LtxB reaction [20]. Although the reaction mechanism of the C-N bond formation was previously discussed in the literature [20, 23], the molecular basis and reaction mechanism of TleB/LtxB have not been identified. Thus, we conducted biochemical analyses with substrate analogs and X-ray crystal structure analyses of TleB and HinD, a protein homolog with 57.5% identity from *Streptoalloteichus hindustanus*, to clarify the reaction mechanism of this unique C(sp2)-H amination and create unnatural indolactams [24]. When we utilized NMVT or *N*-methyl-L-phenylalanyl-L-tryptophanol (NMFT) as a substrate, TleB accepted only NMVT to produce **1** (the label in reference 24) and HinD accepted both NMVT and NMFT to produce indolactams **1** and **4** (Fig. 5). Next, the analogs with N-13 substituted with -NH_2_ (**5**), –OCH_3_ (**6**, **11**), or –OH (**7, 12**) and those with indole ring substitutions with benzo[*b*]thiophene (**13**, **14**) and *N*1-methyl indole (**15**, **16**) were tested (Fig. 5). Interestingly, TleB accepted **5**–**7** and yielded 6/5/6 tricyclic ring compounds (**8**–**10**), which are generated through the reaction between 14-OH and C-2 via the epoxidation between C1-C2. In addition to **9**, TleB also generated the demethylated product **7** from **6**. HinD exhibited broader substrate specificity and oxidized **5**–**7**, **11**, and **12** to produce the tricyclic products **8–10**, **17**, and **18** and the demethylated products **7** and **12**. HinD also produced the *O*-demethylated products **19** and **20** from the benzo[*b*]thiophene substrates **13** and **14**, and the *N*-demethylated products **21** and **22** from the *N*1-methylated substrates **15** and **16**, respectively. These results indicated that N-13 and N-1 should react with the ferryl oxo species (Fe^IV^ = O) in TleB, and their hydrogen atoms should be abstracted to generate the diradical. The TleB structure complexed with NMVT (1.90 Å resolution) and the HinD structure complexed with **13** were solved by X-ray structural analyses (2.35 Å resolution) [24]. Their overall structures adopt the trigonal prism-fold consistently with the P450 oxygenases [25]. In the TleB complex structure, the N-1 of NMVT was closest to the heme iron (5.1 Å), while the N-13 of **13** was closest in the HinD complex structure (4.3 Å) (Fig. 6). These data suggested that the hydrogen atom of NH-1 should be abstracted to generate the first radical and the NH-13 is then abstracted to generate the second radical. When an NMFT analog in which the N-methyl group is substituted with a cyclopropyl group was used as a substrate, we detected the product with NH_2_-13 generated after the reaction of the radical on N-13 with the cyclopropane ring. This result reinforced the hypothesis that the hydrogen atom is abstracted from N-13.

The data shown above supported the diradical pathway in which the P450 generates two radicals from NH-1 and NH-13, and the following radical coupling forms the C-N bond. Firstly, the ferryl oxo species abstracts NH-1, and the subsequent conformational change brings N-13 closer to the ferryl oxo species. The radical that migrated from N-1 to C-4 reacts with the radical on N-13 to form the C-N bond. If the hydrogen bond cannot be abstracted from O-13 or N-13, then the tricyclic compounds are generated via 3β-hydroxy-indolenine formation and ring closure between O-14 and C-2 via epoxidation of the double bond between C-1 and C-2. This study presented beneficial insights on the P-450 catalyzed C-O and C–C radical coupling reactions in nature, such as those in morphine biosynthesis [26].

## Structural analysis of TleC

**Fig. 7:**
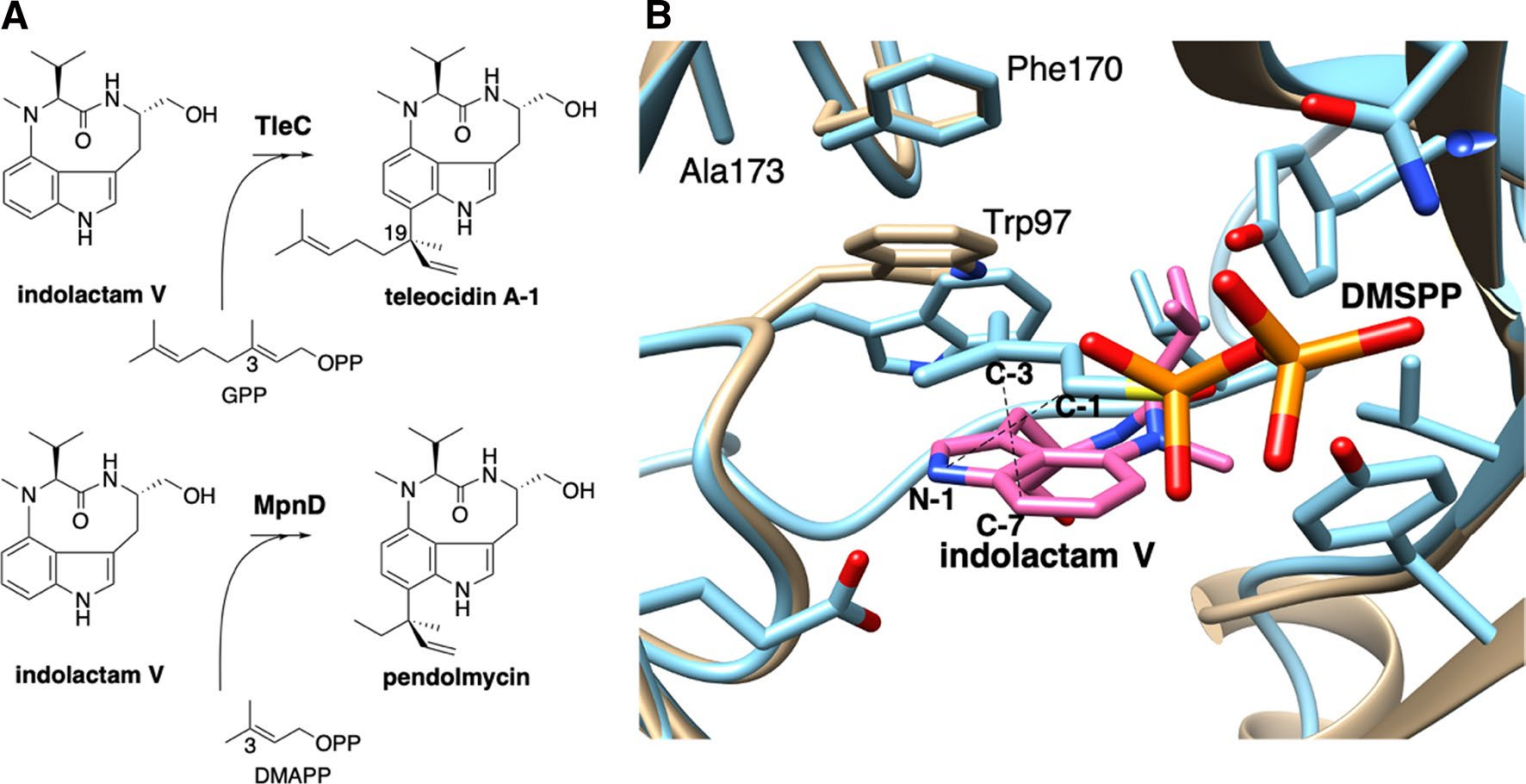
**a** The reactions of TleC and MpnD. **b** The active site structures of TleC. The apo structure (PDB: 4YZK) and the structure complexed with indolactam V and DMSPP (PDB: 4YZL) are colored brown and cyan, respectively

Prenylation increases the lipophilicity of a compound, leading to improved bioactivity in many cases. Prenyltransferase (PT) enzymes catalyze prenylation reactions. Their broad substrate specificity and reactivity are quite interesting to researchers, and this enzyme family has been extensively studied [5, 27–31]. While the C-1 secondary carbocation of the prenyl donor reacts with an acceptor in normal prenylation, the C-3 tertiary carbocation reacts in reverse prenylation, which is rare in nature. TleC catalyzes the reverse prenylation to attach the C10 geranyl moiety on the C-7 of indolactam V, in the same way as LtxC in lyngbyatoxin A biosynthesis (Fig. 7) [14]. To determine the mechanism of C10 reverse prenylation, which had not been identified in any studies, we conducted X-ray crystallization studies of TleC and MpnD complexed with dimethylallyl-S-thiophosphate and indolactam V, and reported their ABBA-fold structures [32] at 2.10 and 1.40 Å resolutions, respectively. MpnD is a homolog of TleC that transfers a C5 dimethylallyl group on the same position of indolactam V [33, 34]. TleC and MpnD share similar overall structures and the indolactam V binding site, but they possess different manners of prenyl chain binding. The C10 alkyl group of geranyl diphosphate (GPP) is retained in TleC through hydrophobic interactions, contributed by Trp97, Phe170, and Ala173. These residues are, respectively, replaced with Tyr80, Trp157, and Met159 in MpnD (Fig. 7). In TleC, Trp97 is flipped by 70º when the substrates are bound to the enzymes, and the cavity volume is increased to accept the C10 alkyl group of GPP. The TleC A173M mutant showed an enhanced preference for dimethylallyl phosphate, and no longer accepted GPP. Interestingly, the TleC W97Y/A173M mutant yielded teleocidin A-2, a C-19 stereoisomer of teleocidin A-1, from indolactam V and GPP, suggesting the potential of enzyme engineering through this mutation to produce compounds with different stereochemistry. In the TleC complex structure, the distance from C-3 of DMAPP to C-7 of indolactam V was 3.3 Å, and is closer than the distance from C-1 of DMAPP to N-1 of indolactam V (6.0 Å) (Fig. 7). These data suggested that the direct C-7 reverse prenylation is more likely than the previously proposed two-step reactions, including N-1 normal prenylation and aza-Claisen rearrangement [12].

## Structural analysis of TleD

**Fig. 8:**
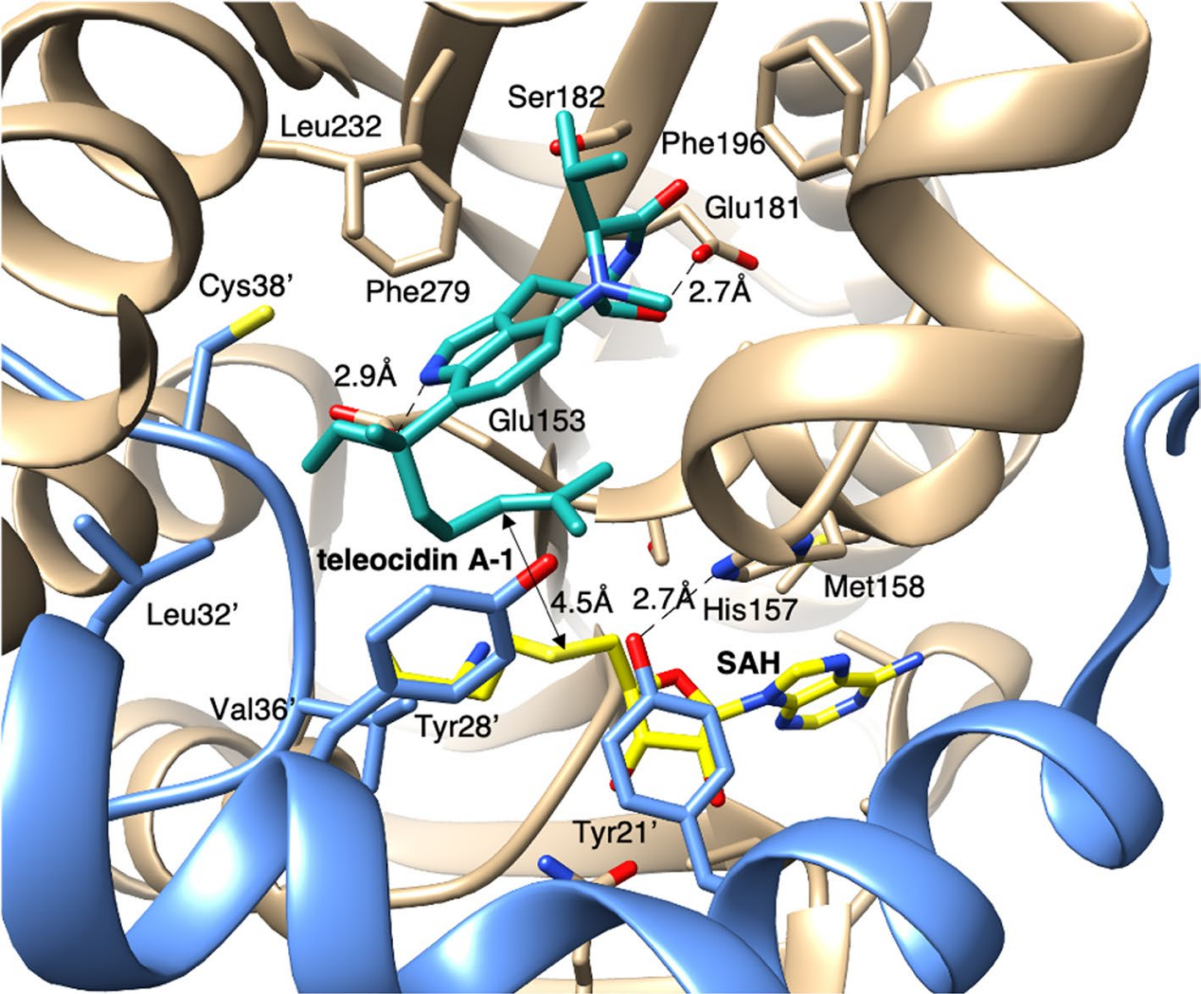
Active site structure of TleD (PDB: 5GM2). Chains A and B are colored brown and blue, respectively

As shown above, the terpene cyclization of teleocidin B was triggered by a methyltransferase TleD [18]. TleD belongs to a family of SAM-dependent methyltransferases which catalyze *C-*, *O-*, and *N-*methylation and affects the polarity of the products [35]. In 2016, Yu et al. reported the 2.80 Å resolution X-ray crystal structure of TleD complexed with *S*-adenosylhomocysteine (SAH) and teleocidin A-1 [36]. The overall structure of its hexamer resembles a typical class I SAM-MT fold [37] with an additional *N*-terminal α-helix. The additional α-helix penetrates into the core of another monomer, and the active site structure is created between them with assistance from the hydrogen bonding between His157 (chain A) and Tyr21′ (chain B) (Fig. 8). In the active site, SAH is bound via a hydrogen-bond network and van der Waals interactions, and teleocidin A-1 is accommodated by hydrophobic interactions with Tyr21′, Tyr28′, Leu32′, Val36′, Cys38′, Ser182, Phe196, Leu232, and Phe279 and hydrogen bonds with Glu153 and Glu181 (Fig. 8). The dihedral angle C23-C24-C25-C26 of teleocidin A-1 is 58º in this model and is consistent with the *Re*-face stereochemistry to produce teleocidin B-4 and des-*O*-methylolivoretin C. The distance between the S atom of SAH and C25 is 4.5 Å, which is reasonable for a *C*-methyltransfer reaction. Their molecular dynamics simulation starting from the one possible conformation showed that the dihedral angle of C23-C24-C25-C26 becomes 60-90º in 1-ns of simulation, thus also supporting the proposed the conformation of telocidin A-1. The N-terminal α-helix is conserved in SpnF, a SAM-dependent methyltransferase-like enzyme that catalyzes [4 + 2] cycloaddition in spinosyn biosynthesis, and it also helps to create the sealed reaction cavity [38, 39]. The hydrophobic nature of the TleD reaction cavity is beneficial to protect the carbocation from an attack by water. This study provided the first structural information on *C*-methylation-triggered terpene cyclization. However, the proposed structural model lacked information on the mechanism by which TleD facilitates the 1,2-hydride shift and arranges the spiro-intermediate to teleocidin B-4 and des-*O*-methylolivoretin C. A detailed kinetic isotope effect calculation and molecular dynamics study would be useful to investigate the more detailed mechanism, as in SpnF studies [40, 41]. Recently, the C-MT from the rhizobacterium *Serratia plymuthica* 4Rx13 was reported to cyclize farnesyl diphosphate to produce pre-sodorifen pyrophosphate [42]. The comparison of this enzyme structure with that of TleD will yield beneficial mechanistic insights.

## Conclusion

This review has summarized the knowledge on the biosynthesis of the terpene indole teleocidin B. The reaction mechanisms, including the C-N bond formation by the P450 oxygenase TleB, the prenyltransfer by TleC, and the terpene cyclization triggered by the methyltransferase TleD, have been discussed in detail, based on the X-ray crystal structures. Currently, their reactions have been analyzed by static structural analyses, but dynamic structural analyses, such as small-angle X-ray scattering, X-ray free-electron laser, and molecular dynamics simulation, would be useful to examine the dynamic structures of these enzymes in the future. Their reaction mechanisms are intriguing from an enzymology viewpoint, and the knowledge is useful for enzyme engineering to create novel medicinal compounds, by using unnatural substrates or heterologous expression systems [43–46]. By complementing the organic synthetic methods [47, 48], more biosynthetic methods to create the analogs will be developed. Among the teleocidin analogs, we will be able to discover useful subtype-specific PKC activators possessing antitumor activities.
